# Photoperiod Affects Node Appearance Rate and Flowering in Early Maturing Soybean

**DOI:** 10.3390/plants11070871

**Published:** 2022-03-24

**Authors:** Nathaniel W. W. Ort, Malcolm J. Morrison, Elroy R. Cober, Bahram Samanfar, Yvonne E. Lawley

**Affiliations:** 1 Department of Plant Science, University of Manitoba, Winnipeg, MB R3T 2N2, Canada; yvonne.lawley@umanitoba.ca; 2 Agriculture and Agri-Food Canada, Ottawa Research and Development Centre, Ottawa, ON K1A 0C6, Canada; malcolm.morrison@agr.gc.ca (M.J.M.); elroy.cober@agr.gc.ca (E.R.C.); bahram.samanfar@agr.gc.ca (B.S.); 3 Department of Biology and Ottawa Institute of Systems Biology, Carleton University, Ottawa, ON K1S 5B6, Canada

**Keywords:** crop plants, floral transition, environmental signals, phenology

## Abstract

The photoperiod plays a critical role in the control of flowering timing in soybean (*Glycine max* (L.) Merr.) with long days increasing the time to flowering. Early flowering cultivars have been developed from breeding programs for environments with long photoperiods; however, this effect is challenging to isolate in field experiments because of other environmental influences. Our experiment examined the effect of photoperiod on the node appearance rate and time to flower for 13 early maturing soybean cultivars ranging in maturity group (MG) between 000.9 and 1.3. Growth chambers were programmed to 14, 15, 16, and 17 h photoperiods and temperature was kept at 25 °C. The date of emergence and main stem node appearance were recorded until flowering. The node appearance rate was slowest for the first node and increased thereafter. All cultivars required more time to flowering in the longer photoperiod treatments and the later rated MG had the greatest sensitivity to photoperiod. A delay in time to flower from a longer photoperiod can delay maturity and expose the crop to fall frost that can reduce seed yield and quality. Understanding and documentation of soybean photoperiod sensitivity will help plant breeders develop suitable cultivars for environments with long photoperiods.

## 1. Introduction

The expansion of early maturing soybean (*Glycine max* (L.) Merr.) into northern growing environments with long photoperiods brings renewed interest in understanding the fundamental relationships between photoperiod and plant development rate (PDR), especially for time to flowering or beginning bloom (R1). Soybean is a quantitative short-day plant and the PDR through vegetative growth to R1 is maximized when a cultivar is grown in a photoperiod less than a critical value or ‘critical photoperiod’. The critical photoperiod varies among cultivars and maturity group (MG) rating [1]. The soybean MG system is used to classify a cultivar based on the duration of time from planting to maturity and range from 10, grown in South America and in the southern USA, to 000, which are grown in northern environments, such as Manitoba [2]. A cultivar assigned an MG greater in value requires more time to reach maturity than a cultivar assigned an MG lesser in value. The MG system is further sub-divided by a decimal grouping which describes the duration to maturity within an MG using the same scale [2]. In general, later MGs are more sensitive to photoperiod than earlier MGs, and later MGs take more time than earlier MGs to reach R1 in long photoperiods [3]. For example, MGs 3 and 5 have been reported to have a critical photoperiods of 13.4 and 12.8 h, respectively [4], and in another study, MGs 2 to 00 were found to have a critical photoperiod of 13.5 h [5]. Photoperiods longer than the critical photoperiod slow PDR by reducing the rate of cell division and specialization, leading to delayed floral induction and evocation or R1 [4,6].

Ten photosensitive genes controlling time to flowering or R1 in soybean have been identified; *E1*, *E2*, *E3*, *E4*, *E6*, *E7*, *E8*, *E9*, *E10*, and *J* [7,8,9]. For all of these genes, except *E6* and *E9*, a dominant allele delays R1 [5,10]. A dominant *E1* has the greatest delaying effect on time to R1 [9] and cultivars rated between MG 000 and 4 typically have an *e1-fs, e1-nl,* or *e1-as* allele [11]. Stewart et al. (2003) [12] reported *E2*, *E3*, and *E4* were similar in their delaying effect while *E1* had approximately double the magnitude of these alleles, and that the effect of these alleles would be additive. Xia et al. (2012a) [13] found cultivars with the *e1-as* allele achieved R1 before cultivars with *E1* but after those with *e1-fs* and *e1-nl*, indicating that the effect of *e1-as* is greater than *e1-fs* and *e1-nl* but less than *E1*.

The effect of photoperiod on the node appearance rate (NdAR) is not as well reported as it is for PDR and is predominantly influenced by air temperature (T) [14]. Additional environmental influences, such as soil moisture, soil fertility, and plant health, further determine NdAR [4,15]. Plant development from emergence (VE, [16]) to R1 is governed by the same environmental influences, and additionally by photoperiod [17]. The NdAR has been described as both consistent and increasing over time. Bastidas et al. (2008) [18] and Tenorio et al. (2017) [14] reported a near-linear relationship between Julian day and NdAR. Conversely, Fehr et al. (1971) [19] described 3–10 days for each of the first three nodes to appear, 3–8 days each for nodes four through six, and 2–5 days for every node thereafter; thus, an increasing NdAR as the plant progressed towards maturity.

We designed a controlled environment experiment to investigate the effect of long photoperiod on the NdAR and PDR from VE to R1 in short-season soybean cultivars with varying MG designations resulting from different combinations of dominant and recessive E genes. Soybean cultivars with later MG and more dominant E alleles were expected to require more time to achieve R1 and will accumulate more nodes prior to R1.

## 2. Results and Discussion

### 2.1. Node Appearance Rate

The number of days from planting to VE was not influenced by photoperiod (*p* = 0.9891) or by an interaction between photoperiod and cultivar (*p* = 0.9624). Soil moisture and soil T were the primary factors influencing seed germination and emergence, and were both favorable for rapid emergence in this experiment. The cultivar CeryxRR took 7.5 days from planting to VE, while the remaining cultivars took 6.3–6.8 days. Seed germination test results for all cultivars were consistently greater than 90%. A visual inspection of the CeryxRR seed lot revealed mold on the seed while the seed for the remaining cultivars were free of mold. This may have led to delayed emergence for CeryxRR. The duration from VE to cotyledon (VC) was consistent among cultivars (*p* = 0.3108) and there were significant differences among photoperiod treatments (*p* < 0.0001). Soybean grown in the 15 and 17 h photoperiod had a shorter duration from VE to VC than the 14 and 16 h photoperiods. Photoperiods of 15 and 17 h may be the optimal level for NdAR during this stage for the T used in this experiment; however, further investigation into this interaction must be performed to confirm this relationship.

The NdAR had significant interactions between cultivar and node number, and photoperiod and node number (Table 1). The cultivar and node number interaction were anticipated as different genetic material, and were likely to vary in response to a similar environment. Further, it was not the objective of this experiment to compare the NdAR of a certain node of one cultivar to a different node of another, and for this reason it was included as a factor in the model to account for variability, but this will not be discussed.

Most cultivars tested in this experiment had consistent NdAR (Table 2). This agrees with Frederick et al. (1989) [20] who found equal NdAR among the cultivars they tested. Sinclair et al. (2005) [21] later reported differences in NdAR among cultivars that had a similar range in NdAR as the current experiment. The consistent NdAR among cultivars in the current experiment may be because of the T used. Grimm et al. (1993) [22] reported optimal T for NdAR in soybean to be 25 °C while Piper et al. (1996) [23] calculated a warmer optimal T from 26 to 30 °C and specified that daily minimum T was a key factor of vegetative growth which was not examined in the current experiment. A greater difference in NdAR among cultivars may have been observed if air T was lower or if T treatments were included. Further studies are recommended to explore T responses including analyzing the NdAR to determine optimal and baseline T for all soybean growth stages.

**Figure 1:**
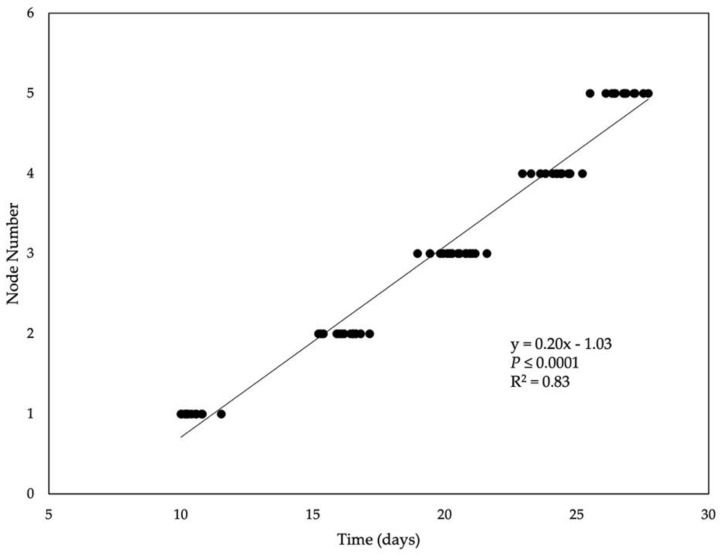
Time from planting (days) to consecutive main stem nodes appearing for soybean cultivars grown under a constant temperature of 25 °C and its corresponding regression equation, coefficient of determination (R^2^), and *p*-value.

The NdAR increased as nodes appeared on the main stem (Table 2). This is consistent with Hesketh et al. (1973) [24] and Fehr and Caviness (1977) [16] who both described a quicker appearance of later main stem nodes than earlier ones. Conversely, Bastidas et al. (2008) [18] and Tenorio et al. (2017) [14] reported a linear relationship between node appearance and time, including among different planting dates in the same experiment. The inconsistent NdAR between these studies may be because of their NdAR analysis. Consistent with Bastidas et al. (2008) [18] and Tenorio et al. (2017) [14], a linear relationship was found in the current experiment between time from planting and node appearance (Figure 1). This relationship, however, does not describe the difference in NdAR among main stem node number but displays node appearance over time. Moreover, in Figure 1, the difference in NdAR between successive nodes is the duration between data points, not the linearity. As the plant progressed towards R1 the time between node appearance became shorter because of the increased NdAR. Additionally, in the previous studies discussed, a greater total number of nodes were recorded and analyzed, contributing to a linear relationship. If only the second to the fifth node were analyzed in these studies, as was in the current experiment, a non-linear trend may have been apparent. In the field, T is increasingly warmer early in the growing season which has been reported to contribute to greater NdAR for later nodes than earlier nodes [14]; however, air T was consistent in this experiment, and therefore the NdAR was unaffected by T among the photoperiod treatments. This suggests the NdAR can be influenced by the growth stage of the plant, but this requires further investigation with different T and photoperiod regimes, as well as among cultivars with varying phenotypic traits.

As photoperiod increased from 14 to 17 h the NdAR decreased from 0.021 to 0.016 1/h of photoperiod (Table 2). This is noteworthy as NdAR has been reported to be unaffected by photoperiod [25,26]. Nico et al. (2015) [27] described a greater number of nodes when natural photoperiod was extended in the field, but a consistent NdAR among photoperiod treatments were still reported. A possible explanation could be that plants grown in the longer photoperiod treatments were exposed to a greater amount of photosynthetic active radiation (PAR), used to produce larger vegetative structures that were not nodes, such as the internode, leaf, or root. These structures require energy and resources to be produced, which could have been diverted from node production, resulting in slower NdAR. Measurements of these structures were not included in this experiment and should be in future studies to test this concept.

**Figure 2:**
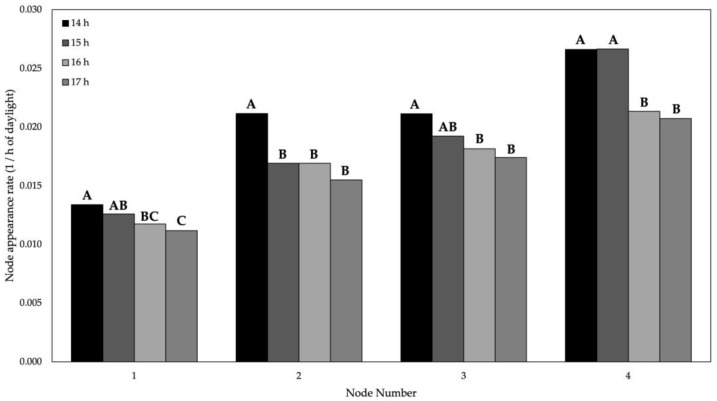
Node appearance rate (NdAR) for nodes 2, 3, 4, and 5 in 14-, 15-, 16-, and 17-h photoperiods. Bars followed by the same letter are not significantly different at *p* < 0.05 according to Tukey’s HSD test for a node.

There was a significant interaction between node number and photoperiod (Table 1). The NdAR for the same node varied among photoperiod treatments and the 14 h photoperiod consistently had greater NdAR than the 17 h photoperiod treatment for all nodes (Figure 2). This may have been for the same reason described previously: a greater supply of PAR favored the development of other vegetative structures over the main stem nodes and resulted in a slower NdAR.

### 2.2. Plant Development Rate and Time to Flowering

Cultivar and photoperiod were significant effects in the ANOVA for PDR (*p* = 0.0001 and <0.0001, respectively) and a significant interaction between them was reported (*p* ≤ 0.0001). The PDR from VE to R1 was then regressed with the photoperiod treatments and the slope, *y*-axis intercept, and coefficient of determination are presented in Table 3. The photosensitivity of a cultivar was determined by the slope of its regression equation in which greater slope equated greater photosensitivity.

**Figure 3:**
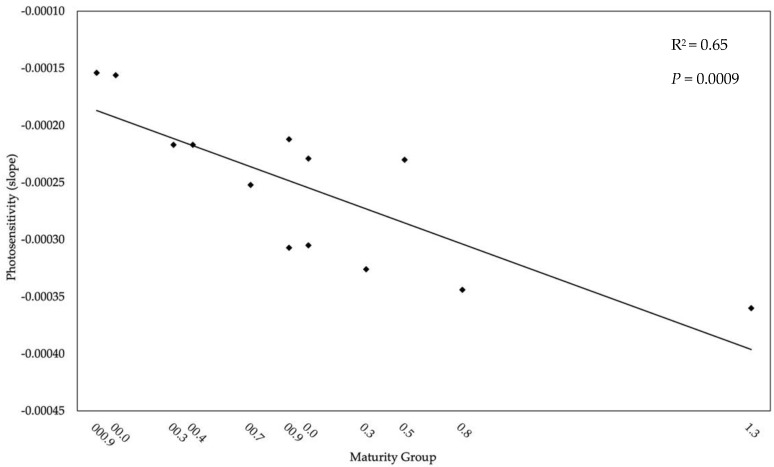
Relationship between increasingly later soybean maturity group (MG) and sensitivity to photoperiod and its corresponding coefficient of determination (R^2^) and *p*-value.

The cultivars tested in this experiment were not isogenic nor near-isogenic lines; however, their sensitivity to photoperiod increased with the number of dominant *E* alleles (Table 3), consistent with previous studies [5,28]. An *e1-nl* or *e1-fs* allele was present in all cultivars except for 9063 which had the *e1-as* allele which has a greater delaying effect on R1 than *e1-nl* and *e1-fs* [13]. This greater effect may have been found if longer photoperiods and additional cultivars or MG were tested. Cultivars with *E2* had a varying degree of photosensitivity and this allele may be insensitive to the photoperiod treatments in this experiment. An *E2* and *E3* allele were present in the two cultivars with the greatest sensitivity to photoperiod and their effect on PDR together may be greater than their individual effect. Cultivars with the greatest sensitivity to photoperiod had an *E3* allele. The recessive *e3* allele has been reported to be insensitive to fluorescent lamps while *E3* remains sensitive [29], explaining why *E3* alleles were in cultivars with the greatest photoperiod sensitivity. *E4* alleles were in cultivars among the range of photoperiod sensitivities determined, indicating they had no effect on PDR under these light conditions and photoperiod durations. This agrees with Cober et al. (1996) [29] who found that *E4* did not delay R1 under either 12 or 20 h photoperiod using a fluorescent light bank similar to the ones used in the current experiment. It is important to note that the ratio of R:FR in the growth chambers of this experiment were not equal to the R:FR of natural light, which likely contributed to the dominant alleles affecting PDR inconsistent to field experiments. The results from this experiment indicate that long photoperiod slows PDR and extends the duration from VE to R1 in early maturing soybean, and when the MG designation increased in value the sensitivity to photoperiod increased (Figure 3), consistent with Major et al. (1975) [3] and Salmerón and Purcell (2016) [30].

### 2.3. R1 Node Number

The mean number of nodes recorded among cultivars at R1 were 4.7, 5.0, 5.2, and 5.4 for the 14-, 15-, 16-, and 17-h photoperiod treatments, respectively. The PDR from VE to R1 was negatively correlated with R1 node number (*p* ≤ 0.0001; r = −0.31), confirming a reduction in main stem nodes at R1 as PDR increased. These results are consistent with Thomas and Raper (1983b) [25] who reported nine more main stem nodes at R1 when soybean was grown in 15 and 16 h compared to 10 and 12 h photoperiods. Câmara et al. (1997) [31] also found soybean grown in a 13 h photoperiod had 1.6 more nodes on the main stem at R1 than soybean grown in a 12 h photoperiod. The results from the current experiment are consistent with the previous literature. When the PDR from VE to R1 is slowed because of a long photoperiod, more nodes are produced prior to and observed at R1. While nodes are an important structure contributing to seed yield, soybean cultivars grown in short environments in North America are typically indeterminate in growth habit and vegetative growth continues post-R1. So, while selecting cultivars with more nodes at R1 for greater seed yield is commendable, the focus for soybean breeders targeting short growing environments should continue to be the timing of R1 and maturity.

## 3. Conclusions

The NdAR and PDR were affected by cultivar, node number, and photoperiod in this experiment. The NdAR was slowest for the first and second node and increased thereafter. The PDR from VE to R1 was slower in the longer photoperiod treatments. In general, cultivars rated to a later MG and cultivars with a greater number of dominant E alleles had a slower PDR and were more sensitive to photoperiod. The slower PDR from VE to R1 resulted in more main stem nodes recorded at R1. As soybean production expands further from the equator into growing environments with long photoperiods it is important to understand and quantify photoperiodism in soybean for developing appropriate cultivars for these new environments. A delay in flowering subsequently results in a longer vegetative growth period which can then delay maturity. This is undesirable if a fall frost occurs prior to physiological maturity, a typical occurrence in short growing environments which can reduce seed yield and seed quality components. This experiment highlights the need for further research exploring the control of flowering in early maturing soybean in long photoperiods until the geographical boundaries of production have been reached for this crop.

## 4. Materials and Methods

Thirteen soybean cultivars ranging in MG rating between 000.9 and 1.3 with E gene combinations of *E2-e2*, *E3-e3*, and *E4-e4*, with a recessive *e1-fs, e1-nl,* or *e1-as* were grown from seed to R1 in four growth chambers. The *E1-E4* maturity loci genotyping were performed using CAP and dCAP allele-specific markers descried by [32] The growth chambers were randomly assigned to 14, 15, 16, and 17 h photoperiods and set to a constant T of 25 °C. The experiment was designed as a Latin square with four replications (four different growth chambers) and each photoperiod treatment was conducted in each growth chamber. Two pots of each cultivar (repetitions) were grown in each replication. The purpose of this experiment was to isolate photoperiod from other environmental variables.

Three seeds were planted approximately 2.5 cm deep in a 2:1 clay:sand mixture in 1.6 L plastic pots. Pots were thinned to one plant at VC and each pot was randomly assigned a number to eliminate observation bias. Prior to filling the pots with soil, a paper coffee filter was placed in the bottom of each pot to prevent soil from leaking through aeration holes at the bottom of the pot. Pots were watered daily and the location of a pot within replications were randomized weekly to ensure equal light and T distribution. Pots remained in the growth chamber until the last pot had reached R1. At the first trifoliate (V1) stage, 50 mL of a modified Hoagland solution containing nitrogen (19.57 mmol L^−1^ of NH_3_-N, 15.59 mmol L^−1^ of NO_3_^−^, 28.31 mmol of CH_4_H_2_O), phosphorus (11.74 mmol L^−1^ of P_2_O_5_), potassium (35.39 mmol L^−1^ of K_2_O), and magnesium and sulfate (2.77 mmol L^−1^ of MgSO_4_ 7H_2_0) was applied to ensure that plants had an adequate source of nutrients and was re-applied every 14 days until R1.

The growth chambers (Enconair Systems Limited©, Winnipeg, MB, Canada) were 2.97 m^2^ and equipped with forty 54 W/840 Min Bipin T5 HO ALTO UNP fluorescent light bulbs (Philips©, Amsterdam, The Netherlands). Light banks were kept 1 m from the top of the plant canopy and were adjusted to maintain a consistent height from the canopy. The ratio of red to far red light was approximately 3.6:0.6 in the growth chambers and was measured using a LI-180 spectrometer (LI-COR Inc., Lincoln, NE, USA). Temperature and *p* were set and controlled using a Honeywell© T775A/B/M Series 2000 Electronic Stand-Alone Controller.

The calendar date of planting, VE, VC, and main stem nodes were recorded on Monday, Wednesday, and Friday until R1. A node was considered to have occurred at the axis of the leaf petiole and the stem, with the first node occurring at the unifoliate leaves and subsequent nodes occurring with the appearance of each successive trifoliate leaf [33]. The first replication in the 16 h photoperiod treatment was eliminated from analysis because of observation error. The sum of illuminated hours that a plant in each replication was exposed to from VE to VC and between subsequent nodes until R1 was calculated by multiplying the photoperiod treatment by the number of days to each stage and the inverse of this was calculated to determine the NdAR or PDR:

NdAR & PDR = 1/Σ illuminated hours(1)

This was not performed from planting to VE because the seedling was below the soil surface during this time and not exposed to photoperiod. Instead, time (in days) was used to measure this period of growth. The NdAR was used to quantify the duration between successive nodes while the PDR was used to describe the development rate from VE to R1.

An analysis of variance (ANOVA) was used to compare treatment means for the NdAR from planting to VE, VE, to VC and node number at R1 using a mixed model and the PROC GLIMMIX procedure of SAS 9.4 (SAS Institute Inc., Cary, NC, USA). Cultivar and photoperiod were fixed effects and replication was treated as a random effect. The data from repetitions were averaged for each replication during statistical analysis.

A repeated measures analysis for main stem nodes 2 to 5 in the PROC GLIMMIX procedure of SAS 9.4 using a normal distribution was used to test the significance of NdAR. An unstructured covariance structure was used, and cultivar and photoperiod were fixed effects. A ‘by statement’ was used to determine cultivar and photoperiod differences in NdAR for each node interval. The NdAR analysis began at V1 to reflect the start of trifoliate node appearance. It was common for more than five nodes to appear prior to R1 in the longer photoperiod; however, statistical analysis was not possible for these nodes because they often did not appear in the shorter photoperiod tested and were recorded as missing data. Therefore, the NdAR analysis concluded at the fifth node or forth trifoliate (V4).

The PDR from VE to R1 was analyzed in a mixed model in the PROC MIXED procedure of SAS 9.4 to evaluate the slope and intercept estimates among cultivars. The growth chamber replications and cultivar repetitions within each replication were random effects and the treatment and cultivar means were separated according to the Tukey–Kramer test with a probability level for significance of 0.05 to control for inflation of family-wise error rate due to multiple testing [34].

## Figures and Tables

**Table 1:** Analysis of variance (ANOVA) for node number, cultivar, photoperiod, and their interaction on the node appearance rate (NdAR) from the second to the fifth main stem node.

Fixed Effect	*p*-Value
Node Number	<0.0001
Cultivar	0.0237
Cultivar × Node Number	0.0003
Photoperiod	<0.0001
Photoperiod × Node Number	0.0023
Photoperiod × Cultivar	0.9827
Photoperiod × Cultivar × Node Number	0.4312

**Table 2:** The node appearance rate (NdAR) from the second to the fifth main stem node for cultivars and their corresponding maturity group (MG), main stem node number, and 14-, 15-, 16-, and 17-h photoperiod treatments.

Fixed Effect		MG	NdAR	
			1/h of Daylight	
Cultivar	Maple Presto	000.9	0.018	ab ^†^
	90A01	00.0	0.019	ab
	Maple Ridge	00.3	0.018	ab
	Alta	00.4	0.019	ab
	Montcalm	00.7	0.018	ab
	Maple Arrow	00.9	0.019	ab
	OAC Carmen	00.9	0.016	b
	AAC Melika	0.0	0.017	ab
	Roland	0.0	0.018	ab
	Rodeo	0.3	0.018	ab
	9063	0.5	0.018	ab
	Dundas	0.8	0.019	ab
	CeryxRR	1.3	0.020	a
Node Number	2		0.012	a
	3		0.018	b
	4		0.019	c
	5		0.024	d
Photoperiod	14		0.021	a
	15		0.019	b
	16		0.017	c
	17		0.016	c

^†^ a–d Least square mean values followed by the same lower-case letter are not significantly different at *p* < 0.05 according to Tukey’s HSD.

**Table 3:** Cultivar, maturity group (MG), slope (development h^−1^ d^−1^), standard error (SE) of the slope, *y*-axis intercept, and coefficient of determination (R^2^) for the plant development rate regressed with photoperiod treatments of 14, 15, 16, and 17 h from emergence (VE) to beginning bloom (R1).

			Regression Equation			Flowering Genotype			
Cultivar	MG	Slope	Slope SE	y-Intercept	R^2^	*E1-e1*	*E2-e2*	*E3-e3*	*E4-e4*
M. Presto	000.9	−0.00015	0.00003	0.00533	0.49	*e1-nl*	*e2*	*e3-tr*	*e4 sore*
90A01	00.0	−0.00016	0.00005	0.00542	0.28	*e1-fs*	*e2*	*e3-tr*	*E4*
OAC Carmen	00.9	−0.00021	0.00003	0.00601	0.66	*e1-nl*	*E2*	*e3*	*e4*
M. Ridge	00.3	−0.00022	0.00003	0.00623	0.59	*e1-nl*	*e2*	*e3-tr*	*E4*
Alta	00.4	−0.00022	0.00004	0.00636	0.59	*e1-nl*	*e2*	*e3-tr*	*e4*
AAC Malika	0.0	−0.00023	0.00004	0.00651	0.60	*e1-fs*	*E2*	*e3*	*E4*
9063	0.5	−0.00023	0.00004	0.00636	0.60	*e1-as*	*E2*	*e3-tr*	*e4 sore*
Montcalm	00.7	−0.00025	0.00004	0.00694	0.62	*e1-fs*	*e2*	*e3-tr*	*E4*
Roland	0.0	−0.00031	0.00003	0.00766	0.74	*e1-fs*	*e2*	*E3*	*E4*
M. Arrow	00.9	−0.00031	0.00004	0.00751	0.71	*e1-nl*	*e2*	*E3*	*e4*
Rodeo	0.3	−0.00033	0.00004	0.00788	0.76	*e1-fs*	*e2*	*E3*	*E4*
Dundas	0.8	−0.00034	0.00004	0.00814	0.77	*e1-fs*	*E2*	*E3*	*e4*
CeryxRR	1.3	−0.00036	0.00004	0.00846	0.80	*e1-fs*	*E2*	*E3*	*E4e4 (H)*

## Data Availability

Not applicable.
